# Evaluation of pharmacy-led weight management service to minimise the risk of cardiovascular disease

**DOI:** 10.1186/s40545-021-00338-3

**Published:** 2021-06-24

**Authors:** Aliki Peletidi, Reem Kayyali

**Affiliations:** 1 Pharmacy Programme, Department of Life and Health Sciences, School of Life Sciences and Engineering, University of Nicosia, Nicosia, Cyprus; 2 Department of Pharmacy, School of Life Sciences, Pharmacy and Chemistry, Kingston University, London, UK

**Keywords:** Pharmacy-led weight management programme, Greek pharmacists’ new clinical role, CVD prevention, Preventive cardiology, Community pharmacy, Public health

## Abstract

**Aims:**

The primary aim of the programme was a minimum of a 5% weight reduction of the initial weight, while the secondary outcomes were a reduction in participants’ body mass index (BMI), waist circumference (WC), blood pressure (BP), AUDIT-C score and an increase in the Mediterranean diet (MD) score and an improvement in physical activity levels.

**Methods:**

This 'before and after' study was a 10-week weight management (WM) programme and it was developed and delivered in community pharmacies in Patras chosen for convenience, thus consisting the first service of its type in Greece. The sample size was calculated (*n* = 96) based on the mean BMI for a Greek male and female individual, and the standard deviation (SD) of weight at baseline of 14 kg.

**Results:**

Nearly every participant enrolled in the 20 participating pharmacies, 97.4% (*n* = 114/117), achieved the programme’s aim, losing at least 5% of their initial weight. The mean percentage of total weight loss of the 117 participants at the 10th week was 8.97% (SD 2.65), and the *t*-test showed statistically significant results (*P*-value < 0.001; 95% CI [8.48, 9.45]). A significant reduction in the waist-to-height ratio (WHtR) was observed in both male (*P*-value = 0.004) and female (*P*-value < 0.001) participants. The participants’ BP and AUDIT-C score and physical activity levels significantly improved (*P*-value < 0.001), as well as their MD score.

**Conclusion:**

This study provides the first evidence that Greek pharmacists have the potential to play an important role within primary healthcare and that after training they are able to provide public health services for both the public’s benefit and their clinical role enhancement.

## Introduction

Weight reduction leads to physical, psychological and social health benefits. According to Vidal et al. [1], 5–10% weight loss brings about beneficial improvements in fasting glycaemia, glycated haemoglobin (HbA1c), both diastolic and systolic blood pressure (BP) and plasma lipid profile. In addition, weight loss can reduce the need to use of glucose lowering medications and minimise the risk of co-morbidities related to type II diabetes [1]. In fact, moderate weight reduction can decrease the total mortality of type II diabetic patients by 25% [1]. MacMahon et al. [2], identified that each Kg lost can achieve an average reduction in systolic and diastolic BP of 0.68 and 0.34 mmHg, respectively (around 1 mmHg overall). Dattilo et al. [3] stated that losing 1 kg can reduce serum cholesterol by 2.28 mg/dl (0.059 mmol/L), low density lipoprotein (LDL) cholesterol by 0.91 mg/dl (0.023 mmol/L), and triglycerides by 1.54 mg/dl (0.017 mmol/L).

Dietary pattern has been shown to influence adiposity and to increase the risk of developing obesity [4]. Measures of obesity include body weight, waist circumference (WC) and body mass index (BMI). However, it is broadly recognised that WC is a better indicator than BMI or weight in terms of identifying the risk of cardiovascular disease (CVD) and/or diabetes [5, 6]. WC is a detector of abdominal obesity. The waist-to-height ratio (WHtR) is also a proxy of visceral (central) obesity [7, 8], which has been newly identified as an “early health risk” detector. According to Ashwell et al. [9], WHtR of < 0.5 is associated with no increased risk; a reading of ≥ 0.5 and < 0.6 is associated with increased risk, while WHtR ≥ 0.6 is associated with a very high risk of adipose obesity.

A cardio-protective diet can help individuals to minimise their risk of developing CVD. A cardio-protective diet mainly includes the consumption of less than 5 g of salt daily, 200 g or more of fruits and vegetables (2–3 portions each), fish (twice a week, one portion of which should be an oily fish such as salmon, sardine, etc.), 30 g (a handful) of unsalted nuts daily and 30–45 g of fibre daily, preferably from wholegrain products [10]. The cardio-protective diet is in line with the Mediterranean diet (MD) [11] consisting primarily of fruit, vegetables, legumes, whole grains, fish and olive oil, with moderate amounts of red meat and alcohol [4, 12].

It has been established that adherence to a MD reduces the risk of developing CVD [13–16]. Sofi et al. [17] stated that MD is a model of healthy eating because of its positive outcomes on an individual’s health. A MD also has a regulating effect on CVD risk factors and conditions including BP, cholesterol levels, insulin resistance, endothelial function and inflammation, although all these effects are linked to an apparent synergy of the entire dietary pattern, rather than being the result of any of its components individually [18, 19]. Therefore, the MD should be considered as a factor with respect to prevention of disease [20].

EMENO project statistics [21] have shown that Greece is in dire need of weight reduction, especially in Patras. There is not only a need to minimise the risks of developing obesity-related medical conditions and CVD, but also to educate the general public to achieve better health outcomes.

Pharmacists are primary healthcare professionals (HCPs) and they have an important role in increasing the public’s awareness about obesity and its consequences. Additionally, based on a resent published report stated that pharmacists in the community have clinical roles over the last 50 years [22]. According to Peletidi et al. [23], Greek pharmacists were found to be eager to expand their role and offer public health services, regarding weight control in particular. There is clear evidence that UK pharmacists and pharmacy staff achieved positive results upon offering CVD prevention public health services including locally commissioned weight management (WM) services to the general public [24]. The WM service offered in the UK could therefore act as a reference for delivering such a service in Greece through pharmacies. The study aimed to design, initiate and evaluate a WM programme offered by pharmacists to the population of Patras in Greece. The primary outcome of the programme was for participants to achieve a minimum of 5% reduction from their initial weight. Secondary outcomes included reduction of BMI, WC, BP, Alcohol Screening Tool (AUDIT)-C score, increase in physical activity and increase in the MD score.

## Methods

This primary care research-based study [25] used mixed research methods [26]. Based on the Toshakkari and Creswell study [27] mixed research methods are described as “research in which the investigator collects and analyses data, integrates the findings and draws inferences, using both qualitative and quantitative approaches and methods in a single study or programme of inquiry”. Mixed research methods can theoretically address a variety of research questions contained in a single study, aimed at exploring the same research issue, but necessitate a number of different research approaches.

### Study design and setting

This ‘before and after’ study designed based on the evidence identified in the UK WM locally commissioned service [24]. Several materials were designed for the purpose of this study. These included three booklets (Initial Screening booklet, Results booklet and Personalised Advice booklet) and the programme’s poster. The Personalised Advice booklet outline was inspired by the “Weigh2Go” service’s manual, a WM service which is currently being offered in Kingston and Richmond LPC in the UK [28].

According to the Pharmaceutical Association of Achaea, there are 223 pharmacies within the municipality of Patras. Pharmacies located in Patras were approached between March and May 2016 and were invited to participate in the pharmacy-led WM programme (via e-mail, telephone, personal contact). Thirty pharmacists expressed an interest in participating in the programme with 26 pharmacists finally actually taking part in the programme.

The required minimum sample size to show statistical significance was 96 participants. The minimum sample size was calculated based on a 5% weight loss pre- and post-intervention using the paired *t*-test sample size calculation (using a significance level of 0.05 for two sided tests) for before–after study, in accordance with previous studies [29, 30]. The sample size calculation used was based on the standard deviation of weight at baseline (initial screening) which was 14 kg (14 kg is the difference between two BMI classifications, e.g. overweight and obese I) and on the difference in mean weight of 4 kg, based on a 5% weight reduction. This calculation used online calculators based on Rosner et al. [31, 32]. To consider drop-outs, if participants dropped out halfway through the programme (5 weeks), they could be replaced.

All relevant documentations were ethically approved by the Pharmaceutical Association of Achaia (Ref: 56), the Sub-Mayoralty for Health Social Policy and Welfare of the Municipality of Patras (Ref: 115/32617) (March 2016) as well as by the Science Engineering and Computing (SEC) Ethics Committee (ID: 1516/030) in Kingston University (KU) London (May 2016). All materials were validated with a Greek pharmacist (member of the Pharmaceutical Association of Achaia) with no major changes recommended. Participants information sheets (PIS) including all the necessary information were provided before programme’s delivery to both providers (pharmacists) and receivers (participants). Additionally, they contained a consent form to be completed by the pharmacists and the participants, respectively.

The programme was advertised through posters placed on display in participating pharmacies or by the pharmacists themselves.

### Intervention

The programme comprised 11 (including the initial screening) consecutive visits. Enrolment on the programme was made upon completion of initial screening to ensure participants met the eligibility criteria (adult men and women (≥ 18 years old) without any diagnosed risk conditions such as diabetes, hypertension and dyslipidaemia, or any CVD, or cancer or any other chronic conditions, BMI ≥ 25 and ≤ 35 kg/m^2^ unless a cardiologist confirmed their eligibility to participate, those without specific dietary habits, such as vegetarians, vegans, or those who have special dietary requirements such as a gluten-free regime and those with no food allergies and/or food intolerance, anyone who could freely give consent without language barriers, mental capacity, or learning disability. Candidates who met the inclusion criteria were then enrolled in the 10-week programme.

Initial screening included a series of assessments to enable pharmacists to identify if the participant was eligible for the programme. Initially, demographic information (age, gender, educational status and monthly income) was taken. Medical lifestyle assessments (individual's smoking status, alcohol status and eating status (their typical diet, the number of meals/snacks they consume) were also taken. An important aspect of this WM programme was that it was the first study assessing other important weight gain contributors including alcohol intake (by using a shorter version of the AUDIT score (AUDIT-C)), and adherence to the MD (based on participants’ answers to 14 questions) [11]. Based on Traversy et al. [33], 1 g of alcohol gives 7.1 kcal (29 kJ) and in conjunction with the energy acquired from other dietary sources can promote weight gain. The MD score was an essential addition to the programme as an improvement of 2 points in a MD score was found to be associated with a decrease of 9% in overall mortality, 9% in CVD-related mortality [17] and it can help in weight reduction [34]. The programme also included a BP measurement. Although there are previous studies assessing BP within a pharmacy-led weight management service [35–37], most of them did not report their findings [35, 36].

Eligible participants who entered the programme were assigned a “Results” booklet and received a “Personalised Advice” booklet and a “Meal Planner”. As part of the programme participants were asked to maintain food and exercise diaries weekly, as well as to record their weekly weight measurements. A diary was provided in the booklet for participants to fill out.

On each visit, pharmacists measured participants’ weight, WC, BP and HR, and also assessed participants reported physical activity levels. In addition, they calculated their BMI, and determined whether participants had reduced or increased their body weight, their BMI or WC. Through this, pharmacists were able to identify whether the programme had affected participants positively or negatively.

The frequency of the visits (biweekly) and the duration of the service were planned based on the experience of UK pharmacists and pharmacy staff who offer weight management service in the UK [24]. Details of the visits are described below (Table 1).

**Table 1:** Details of the visits included in the weight management programme

Visit 1 (initial screening)	Visit 2	Visit 3	Visit 4	Visit 5	Visit 6
Week 1	Week 3	Week 5	Week 7	Week 9	Week 11
If eligible for the programme: - distribution of the “personalised advice” and results booklets - goal setting - European Society of Cardiology (esc) healthy diet characteristics and exercise recommendations (2016 esc guidelines on CVD prevention in clinical practice) [10]	Importance of healthy-balanced diet and portion size	Importance of physical activity	Alcohol and weight, Food labelling	Shopping tips and eating out	How to maintain the weight loss - discussion of achievements - completion of service’s evaluation questionnaires - further follow-up if requested
Monitor parameters Monitoring period (visit 2–5): weight, WC, BMI, BP, HR, weight loss/gain between visits, physical activity levels Final visit (visit 6): weight, WC, BMI, BP, HR, final audit-c score, % total weight loss, % total wc loss, % total bmi alteration					

*BMI* body mass index, *BP* blood pressure, *CVD* cardiovascular disease, *WC* waist circumference, *HR* heart rate

### Data collection

The data recorded by pharmacists were collected at the end of the programme in January 2017 in person. Additionally, the researcher collected participants’ weight either self-reported or measured by the pharmacists in October 2017 (9–12 months after the programme’s termination depending on each participant starting date). The programme adhered to the Common Law Duty of Confidentiality via the Data Protection Act 1998.[38, 39] Pharmacists kept all participants’ details and data, including consent forms, in a separate folder for each participant, maintained in a securely locked drawer or cabinet until the programme’s end. All Excel spreadsheets were kept in encrypted and password-protected files on a computer, so that only they would have access to them. After the termination of the programme, all collected data including participants’ measurements as well as participants’ and service providers’ consent forms were provided to the researcher by the pharmacists. For pharmacies that used the Excel spreadsheets to record results, the completed Excel spreadsheets were saved to the researcher’s USB flash drive to ensure confidentiality of the transferred data. All the anonymised data was collected either in hard copies (booklets) or in Excel were stored in a secure place in a locked cabinet/password protected file at KU. Participants were coded to maintain anonymity based on the number of the pharmacy which provided them with the programme, and in numerical order. For example: Weight Management (WM): WM1_1, WM3_12, etc.

### Data analysis

All data collected from each of the participating pharmacies were entered into a Microsoft Excel 2010 spreadsheet and then, they were exported into IBM Statistical Package for Social Sciences (SPSS) Version 23 for statistical analysis. The results are expressed as mean differences with 95% confidence intervals (CIs) and *P*-values. CIs not including zero and *P*-values less than 0.05 were considered as statistically significant.

The programme was considered successful if a participant achieved at least a 5% reduction in their initial weight, which was the stated goal of the programme. To test if the participants’ total percentage weight loss was ≥ 5%, the one-sample *t*-test was used. Additionally, to examine participants’ weight change between the last visit and the follow-up period (after 9–12 months of the programme’s termination), a one-sample *t*-test was conducted. Furthermore, to test the effect of the programme on participants’ BMI, a paired-samples *t*-test was used comparing participants’ BMI at the first visit (initial screening) and the last visit (programme completion). In addition, an unpaired samples *t*-test was conducted to assess the impact of gender on BMI reduction.

According to NICE guidelines [40], the classification of WC as low, high, or very high is different between males and females. Therefore, the effect of the programme on participants’ WC was tested separately for male and female participants using the paired-samples *t*-test. Moreover, the effect of gender (female/male) on WC reduction was tested using the unpaired samples *t*-test. The WHtR, as mentioned previously, is a proxy of visceral (central) obesity [7, 8], which has been newly identified as an “early health risk” detector. To examine the programme’s effect on reducing participants WHtR the McNemar’s test was utilised (per gender). Furthermore, in order to examine the effect of programme participation on improving BP status (BP readings were stratified into three groups: 1 = optimal + normal, 2 = high normal, 3 = hypertensive) the Sign test was conducted.

Additionally, to test the effect of the programme on participants’ exercise habits, a paired-samples *t*-test was conducted comparing participants’ exercise reported at the first visit (initial screening) and the last visit (programme completion). In order to examine if the changes (increase/decrease) of mean exercise time per visit during the programme (i.e. the participant spent the same/less time or more time for exercise than the previous visit) affected the changes (increase/decrease) in weight loss (i.e. the participant had the same/less weight loss or more weight loss than the previous visit) a Chi-square test was conducted.

As stated above, an increase in the MD score of 2 points is related to a 9% decrease in CVD incidence and mortality [11, 4]. Therefore, in order to examine if participation in the WM programme had at least a 2-point increase in the MD score, a one-sample *t*-test was conducted. Finally, the programme’s effect on participants’ alcohol consumption habits was also assessed. The AUDIT-C score was calculated by the pharmacists at both the initial screening and the last visit. To test the aforementioned effect, the paired-samples *t*-test was utilised.

## Results

### Pharmacists

Of the 26 pharmacists who agreed to participate and deliver the WM programme, only 21 pharmacists were able to enrol participants. The demographics of the 21 pharmacists who recruited participants are described in Table 2 below.

**Table 2:** Personal and practice demographic information of pharmacists who recruited individuals (*n* = 21)

Gender distribution		
Sex	Frequency (*n*)	Percentage (%)
Males	12	57.1
Females	9	42.9
Age distribution		
Age range	Frequency (*n*)	Percentage (%)
25–34 years	6	28.6
35–44 years	10	47.6
45–54 years	3	14.3
55–69 years	2	9.5
Years of experience		
Year range	Frequency (*n*)	Percentage (%)
≤ 5 years	6	28.6
6–10 years	5	23.8
11–15 years	4	19
16–20 years	2	9.5
≥ 20 years	4	19

The mean total time a pharmacist spent for all six sessions was 151.43 min (SD = 25.84) (approximately 2 h and 30 min) with a range from 97.5 to 198.75 min

### Analysis of participants’ measurement data

A total of 119 individuals, living in Patras, expressed an interest in enrolling onto the programme. However, only 117 participants completed the informed consent form to take part in the study. All enrolled participants completed the 6 biweekly visits (including the initial screening). None of the participants requested a further follow-up, which was an option after completion of the core 10-week monitoring period. The demographics of the sample taken at the initial screening are described in Table 3.

**Table 3:** Characteristics of the participants at initial visit (*n* = 117)

Gender distribution	Sex			Frequency (*n*)		Percentage (%)			
	Males			32		27.4			
	Females			85		72.6			
Age distribution	Age range			Frequency (*n*)		Percentage (%)			
	18–24 years			6		5.1			
	25–34 years			18		15.4			
	35–44 years			29		24.8			
	45–54 years			32		27.4			
	> 55 years			32		27.4			
Educational level	Type			Frequency (*n*)		Percentage (%)			
	Primary school			3		2.6			
	Secondary school			49		41.9			
	Higher education (college/university)			65		55.6			
Monthly income	Euros (€)			Frequency (*n*)		Percentage (%)			
	< 500			26		22.2			
	500–999			53		45.3			
	1000–1500			22		18.8			
	> 1500			7		6.0			
	No reported income			9		7.7			
Smoking status *Pack years can be calculated as follows: number of pack-years = number of cigarettes smoked per day/20) × number of years smoked	Smokers			Frequency (*n*)		Percentage (%)			
	No			84		71.8			
	Yes			33		28.2			
	Pack years*			Frequency (*n* = 33)		Percentage (%)			
	≤ 15			13		39.4			
	> 15			20		60.6			
	Have you ever thought to stop smoking? (*n* = 33)								
	Answer			Frequency		Percentage (%)			
	Yes			28		84.8			
	No			5		15.2			
	Do you have concerns that when you quit smoking you will gain weight? (*n* = 33)								
	Answer			Frequency		Percentage (%)			
	Yes			25		75.8			
	No			8		24.2			
AUDIT-C score	Scores			Frequency (*n* = 80)		Percentage (%)			
	1			17		21.3			
	2			14		17.5			
	3			13		16.3			
	4			10		12.5			
	5			8		10			
	6			9		11.3			
	7			5		6.3			
	8			1		1.3			
	9			1		1.3			
	10			2		2.5			
Eating status	Typical diet					Frequency (*n*)			Percentage (%)
	Mainly vegetables					2			1.7
	Mainly meat (including poultry and fish)					12			10.3
	Mainly meat (including poultry and fish) and vegetables					12			10.3
	Variety of foods (e.g. fruits, vegetables, meat, dairy sweets, fatty and starchy foods)					91			77.8
	Reported meals/day					Reported snacks/day			
	Number of meals		Frequency (*n*)	Percentage (%)		Number of snacks	Frequency (*n*)		Percentage (%)
	1		8	6.8		0	39		33.3
	2		56	47.9		1	28		23.9
	3		38	32.5		2	29		24.8
	4		11	9.4		3	11		9.4
	5		4	3.4		4	9		7.7
						> 4	1		0.9
	Mediterranean diet score					Frequency (*n*)			Percentage (%)
	≤ 5					28			23.9
	6–7					47			40.2
	8					19			16.2
	9					13			11.1
	≥ 10					10			8.5
Physical activity (PA)		No reported PA (participants)			PA < 150 min (participants)			PA ≥ 150 min (participants)	
	Frequency (*n*)	28			38			51	
	Percentage (%)	23.9			32.5			43.6	
	Mean minutes	-			81.71 (SD = 30.5)			248.53 (SD = 82.7)	
	Minimum minutes				20			150	
	Maximum minutes				130			540	

A comparison between the baseline data collected at the initial screening (1st visit) and that collected at the end of the programme (last visit 6th visit) was conducted to identify the effect of the pharmacy-led weight management programme on participants’ total weight, BMI and WC. The results are summarised in Table 4. In addition, a comparison was also made after 9–12 months’ follow-up in participants’ weight results.

**Table 4:** Weight Loss, % total weight loss, BMI and WC outcomes of the 10-week weight management programme (*n* = 117)

Weight								
Weight measured in each visit (kg)	1st visit	2nd visit	3rd visit	4th visit		5th visit	6th visit	
Mean	86.98	85.25	83.77	82.45		80.97	79.24	
SD	16.85	16.47	16.30	16.27		16.05	15.93	
Minimum	57	57	57	57		55.7	53.5	
Maximum	155	153	150	148		146	143.5	
Mean weight loss between visits	1st to 2nd visit	2nd to 3rd visit	3rd to 4th visit	4th to 5th visit		5th to 6th visit		
	− 1.72	− 1.49	− 1.32	− 1.48		− 1.72		
Total mean weight loss (kg)	7.73, SD 2.51		Minimum total weight loss (Kg)		Maximum total weight loss (Kg)			
			0		16.5			
Weight alteration between visits	1st to 2nd visit	2nd to 3rd visit	3rd to 4th visit	4th to 5th visit		5th to 6th visit		
Frequency of participants (*n*)/Percentage of participants (%)								
Weight loss	110/94	112/95.7	107/91.5	110/94		117/100		
No change	7/6	5/4.3	8/6.8	3/2.6				
Weight gain	-	-	2/1.7	4/3.4				
% total weight loss achievements	Frequency (n)	Percentage (%)	Minimum % total weight loss	Maximum % total weight loss		Mean % total weight loss		SD
< 5%	3	2.6	0	16		8.97		2.65
5–10%	59	50.4						
> 10%	55	47.0						

BMI							
BMI measured in each visit (kg/m^2^)	1st visit	2nd visit	3rd visit	4th visit	5th visit	6th visit	
Mean	31.14	30.52	29.99	29.51	28.99	28.36	
SD	4.34	4.24	4.23	4.24	4.20	4.18	
Minimum	25.08	24.56	24.14	23.73	23.31	22.48	
Maximum	47.84	47.22	46.30	45.68	45.06	44.29	
Mean BMI loss between visits	1st to 2nd visit	2nd to 3rd visit	3rd to 4th visit	4th to 5th visit	5th to 6th visit		
	− 0.6151	− 0.5314	− 0.4788	− 0.5272	− 0.6235		
BMI alterations between visits	1st to 2nd visit	2nd to 3rd visit	3rd to 4th visit	4th to 5th visit	5th to 6th visit		
Frequency of participants (n)/Percentage of participants (%)							
BMI loss	110 /94	112/95.7	108 /92.3	110/94	117/100		
No change	7/6	5/4.3	7/6	3/2.6			
BMI gain	–	–	2/ 1.7	4/ 3.4			
WC							
WC measured in each visit (cm)	1st visit	2nd visit	3rd visit	4th visit	5th visit		6th visit
Mean	106.49	104.83	103.08	101.68	100.31		98.68
SD	15.00	14.13	14.09	14.04	13.89		13.86
Minimum	79	78	76	75	74		73
Maximum	155	147	149	148	144		144
Mean WC loss between visits	1st to 2nd visit	2nd to 3rd visit	3rd to 4th visit	4th to 5th visit	5th to 6th visit		
	− 1.66	− 1.75	− 1.40	− 1.36	− 1.64		
WC changes between visits	1st to 2nd visit	2nd to 3rd visit	3rd to 4th visit	4th to 5th visit	5th to 6th visit		
Frequency of participants (n)/Percentage of participants (%)							
WC reduction	88/75.2	94/80.3	94/80.3	98/83.8	106/90.6		
No change	21/17.9	17/14.5	17/14.5	16/13.7	10/8.5		
WC increase	8/6.8	6/5.1	6/5.1	3/2.6	1/0.9		

Note that values with (-) sign shows weight loss and values with ( +) sign shows weight gain

The mean weight was reduced from 86.98 kg (SD = 16.47) at the initial screening to 79.24 kg (SD = 15.94) at the sixth (last) visit. The mean total weight loss was 7.73 kg (SD = 2.51) at the last visit. The primary outcome for the programme was for participants to achieve at least a 5% weight reduction on completing the programme. Nearly every participant (97.4%, (*n* = 114) achieved the programme’s aim. More specifically, 50.4% (*n* = 59) lost between 5 and 10% of their initial weight and 47% (*n* = 55) lost ≥ 10% of their initial weight while 3 participants (2.6%) did not achieve the aim of the programme (Table 4). The mean percentage total weight loss of the 117 participants at the 10th week was 8.97% (SD 2.65) (minimum % total weight loss 0 and maximum % total weight loss 16) (Table 4), and the *t*-test showed statistically significant results (*P*-value < 0.001; 95% CI [8.48, 9.45]), proving that the programme was successful. Furthermore, participants were asked to either self-report or to visit the pharmacies in which they had participated in the weight management programme, to measure their weight at 9–12 months follow-up. Of 117 individuals who participated into the programme, 111 (94.9%) either self-reported or had their weight measured by their pharmacist to identify if they were maintaining their weight loss. The analysis indicated that almost an equal number of participants either lost (*n* = 49) or maintained their weight (*n* = 48) compared with the measured weight at the last visit. Only a minority (n = 14) gained weight (Table 5). More specifically, of those who lost further weight, 18.8% (*n* = 22) of participants lost ≤ 1 kg and 23.1% (*n* = 27) between 1 and 5 kg (Table 5). Additionally, of those participants who gained weight, 5.1% (*n* = 6) gained ≤ 1 kg, 6% (*n* = 7) gained between 1 and 5 kg and only 0.9% (*n* = 1) gained > 5 kg. In terms of the total percentage of weight loss in the follow-up, more than half of the sample (45.3%, *n* = 53) still achieved more than 10% weight loss and 42.7% (*n* = 50) achieved between 5 and 10% total weight loss. Only 6.8% had a total percentage weight loss of below 5% (Table 5).

**Table 5:** Self-reported or measured weight at 9 to 12 months after termination of the programme (*n* = 111)

Weight self-reported/measured (kg) after 9–12 months	
All participants (*n* = 111)	
Mean	79.275
SD	16.15
Minimum	53.5
Maximum	142.0
Males (*n* = 32)	
Mean	92.747
SD	17.40
Minimum	62.0
Maximum	142.0
Females (*n* = 79)	
Mean	73.818
SD	11.9613
Minimum	53.5
Maximum	109.0

Changes in weight (comparison between last visit and follow-up period)	Frequency (*n*)	Percentage (%)
Weight loss (*n* = 49)		
Weight loss ≤ 1 kg	22	18.8
Weight loss 1– ≤ 5 kg	27	23.1
Weight gain (*n* = 14)		
Weight gain ≤ 1 kg	6	5.1
Weight gain 1– ≤ 5 kg	7	6.0
Weight gain > 5 kg	1	0.9
No change between the weight measured at the last visit (n = 48)		
No change	48	41.0

% total weight loss after 9–12 months follow-up (compared to initial visit)	Frequency (*n*)	Percentage (%)
0–5%	8	6.8
5–10%	50	42.7
> 10%	53	45.3
Males (*n* = 32)		
0–5%	3	9.4
5–10%	16	50.0
> 10%	13	40.6
Females (*n* = 79)		
0–5%	5	5.9
5–10%	34	40.0
> 10%	40	47.1

Furthermore, a one-sample *t*-test showed that the mean percentage of weight change between the last and the follow-up visit (mean = -0.57, SD = 1.79) was statistically significant (*P*-value < 0.001)

## BMI achievements

Regarding participants’ BMI, it was observed that the mean BMI was continuously reduced during visits from 31.14 kg/m^2^ at the first visit (SD = 4.34) to 28.36 kg/m^2^ at the sixth visit (SD = 4.18) (Table 4) as BMI is related to weight reduction.

In terms of the participants’ BMI achievements, at the end of the 10-week programme (last visit), 27 (23.1%) of the participants achieved a normal BMI, *n* = 56 (47.9%) were overweight, 25 (21.4%) were obese class I, 7 (6%) obese class II, and 2 (1.7%) obese class III.

Participants (27 out of 56) who were enrolled in the programme as “overweight (25–29.9 kg/m^2^)” achieved a “normal (18.5–24.9 kg/m^2^)” BMI at its end. Additionally, 27 out of the 41 participants who were initially classified as “obese class I (30–34.9 kg/m^2^)” shifted to “overweight (25–29.9 kg/m^2^)”, 11 out of 14 participants who initially classified as “obese class II (35–39.9 kg/m^2^)” remained obese but shifted to “obese class I (30–34.9 kg/m^2^)”, and 4 out of 6 who initially classified as “obese class III (≥ 40 kg/m^2^)” reached “obese class II (35–39.9 kg/m^2^)”.

A paired-samples *t*-test was conducted to evaluate the impact of the programme on participants’ BMI. There was a statistically significant decrease in BMI between the initial visit (mean = 31.14, SD = 4.34) and the last visit/end of the programme (mean = 28.36, SD = 4.18); *t* (116) = 36.01, *P*-value < 0.001). Additionally, there was no statistically significant difference between males (mean = 2.63, with SD = 0.93) and females (mean = 2.83 with SD = 0.79), *P*-value = 0.234.

After 9–12 months follow-up it was observed that of the 111 participants who either self-reported or visited the pharmacist to measure their weight, 104 participants remained in the same BMI category and 4 participants improved their BMI classification (2 participants from overweight moved to normal weight, 1 from obese class I to overweight and 1 from obese class II to obese class I). Interestingly, 3 participants moved from a better BMI category to a worse one. More specifically, 1 participant moved from normal BMI to overweight, 1 participant from overweight to obese class I and 1 participant from obese class I to obese class II.

## Waist circumference achievements

In terms of gender, the WC decreased from 116.59 cm, (SD = 13.40) at the first visit to 108.58 cm (SD = 12.69) at the sixth visit for male participants. For females, the decrease was from 102.68 cm (SD = 13.81) at the first visit, to 94.95 cm (SD = 12.42), at the sixth visit. At the end of the programme, a number of male participants (7/32) not only reduced their WC but improved their WC classification. Six male participants shifted from a classification of very high to high, and 1 participant from high to low. The mean value of the total WC reduction for males was 8.02 cm (SD = 3.01) (95% CI [6.93, 9.10]). The programme’s impact on males’ WC was tested using a paired-samples *t*-test. It was identified that there was a statistically significant decrease in WC between the first (mean = 116.59, SD = 13.40) and last visit (mean = 108.58, SD = 12.69), *P*-value < 0.001.

In addition, almost 1 in 4 women (21/85) positively changed their WC classification. More specifically, 7 women participants shifted from high risk to low risk and 14 from very high risk to high risk. The mean value of the total WC reduction in females was 7.73 cm (SD = 4.14), (95% CI [6.84, 8.62]). A paired-samples *t*-test showed a statistically significant decrease in females’ WC between the first (mean = 102.68, SD = 13.82) and last visit (mean = 94.95, SD = 12.42), *P*-value < 0.001.

Finally, an independent samples *t*-test was used to test the effect of gender on the WC reduction showing no statistically significant difference between males (mean = 8.02, SD = 3.01) and females (mean = 7.73, SD = 4.14), *P*-value = 0.723.

## Correlation between BMI and WC in identifying CVD risk

According to NICE guidelines [40], the correlation of BMI to WC can indicate health risks related to being overweight or obese. According to the male participants’ results, 19 were found at the initial screening to be “very high risk”, 10 “high risk”, 2 “increased risk” and only 1 participant with “no increased risk”. The pharmacy-led weight management programme was not only successful in reducing weight, BMI and WC, but also helped to decrease the health risks associated with being overweight and obese. After the programme, a decrease in the number of male participants with a “very high risk” was observed, from 19 to 11 individuals. Additionally, an increase in male participants with “no increased risk” (from 1 to 5 males), with “increased risk” (from 2 to 4) and in participants with “high risk” (from 10 to 12) was observed (Table 6).

**Table 6:**
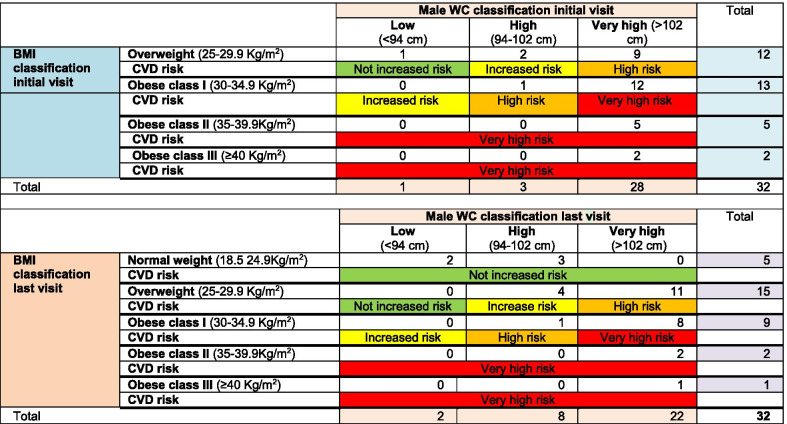
Correlation between the BMI, WC and the CVD risk at the Initial and last visit for male participants

In terms of female participants before the programme delivery, 2 females were found to have “no increased risk”, whereas after the programme delivery this number increased by 24 participants (total 26 females). Additionally, 9 females were identified as having an “increased risk” before the service, whereas after the service 10 females were found to be at “increased risk”. The next important change was that of 34 females who were found to be at “high risk” after the service delivery, the number of participants in this category was reduced to 27. Finally, before the programme delivery there were 40 females at “very high risk”, but after the programme, the number of female participants in this category was reduced to almost half (*n* = 22) (Table 7).

**Table 7:**
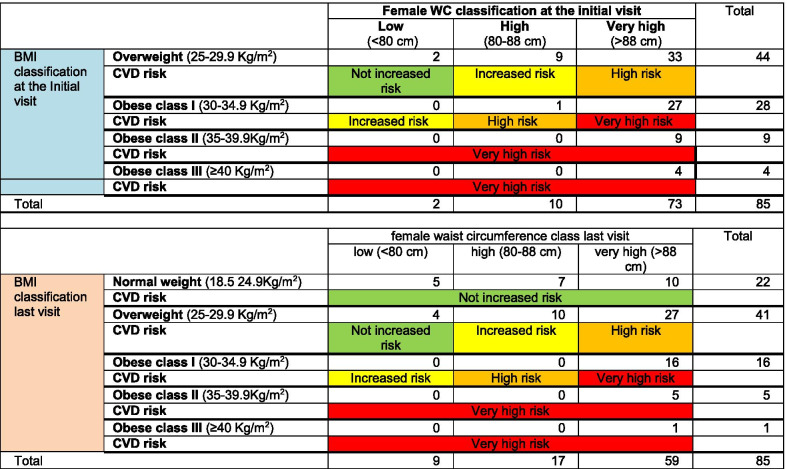
Correlation between the BMI, WC and the CVD risk at the Initial and last visit for female participants

## WHtR and abdominal obesity

After the programme’s termination almost a quarter of participants (25%, *n* = 8 males and 22.3%, *n* = 19 females) moved from a WHtR ≥ 0.6 to a WHtR < 0.6.

In order to examine the effect of the programme on reducing the WHtR, of males from (WHtR ≥ 0.6 to WHtR < 0.6) the McNemar’s test was conducted. The effect of the programme was statistically significant (*P*-value = 0.004). Similarly, the reduction of WHtR for female participants from WHtR ≥ 0.6 to WHtR < 0.6 was statistically significant (*P*-value < 0.001).

## Physical activity achievements

The majority of participants (54.7%) were reported to exercise more than 150 min per week during the programme (mean = 230.94, SD = 83.06) compared to 43.6% (mean = 248.53, SD = 82.7) at start of the programme. Even if participants showed an increase in their physical activity from initial screening to the last week, the results did not indicate any statistically significant difference between the mean percentage of total weight loss between group 1 (participants who exercised less than the recommended 150 min weekly) (mean = 8.91, SD = 3.05) and group 2 (participants who exercised equal or more than the recommended 150 min weekly) (mean = 9.02, SD = 2.29), *P*-value = 0.829.

## BP achievements

In order to examine if participants’ BP status was improved through the programme, three groups were generated: optimal and normal BP, high normal BP, and hypertensive. Sixteen participants out of 19 who were initially identified with hypertension moved to optimal- normal or high normal levels, with only 3 remaining in the hypertensive category after the programme’s termination. The Sign test was conducted showing statistically significant results (*P*-value < 0.001) between the initial BP classification and the last visit.

## Effect of the programme on the AUDIT-C score

The weight management programme also contributed to the AUDIT-C score reduction of the participants. After the WM programme, none of the participants identified with an AUDIT-C score between 8 and 10, compared with the initial screening scores. More specifically, 83.8% (*n* = 67/80) of the participants decreased their AUDIT-C score after the programme’s conclusion. The reduction is participants’ alcohol consumption as measured by the AUDIT-C score was found to be statistically significant (mean AUDIT-C at end of programme = 1.86, SD = 1.59, mean AUDIT-C at start of programme = 3.63, SD = 2.29, *P*-value < 0.001).

## Mediterranean diet and WM programme

The MD score was also calculated for each participant at the initial and the last visit. It is obvious that after the programme’s delivery there was an increase in the mean MD score. Additionally, there was an increase in participants whom their score was greater than or equal to 10 (*n* = 10, 8.5% at initial screening and *n* = 33, 28.2% at the last visit). The high score indicates that better adherence to the MD was achieved. A paired-samples *t*-test showed that the mean MD score taken at the last visit (mean = 8.33, SD = 1.99) is statistically significantly greater than the mean MD score at the initial screening; *t* (116) = -13.99, *P*-value < 0.001.

As mentioned previously, an increase of 2 points in the MD score is related to a 9% decrease in CVD risk [4, 11]. Interestingly, after the end of the programme, 44 participants (37.6%) increased their Mediterranean score by 1 point and 48 participants (41%) increased it by at least two. However, the one-sample *t*-test showed that the mean increase in Mediterranean score (mean = 1.49, SD = 1.15) was not statistically significant; *t* (116) = -4.826, *P*-value = 1.

## Discussion

This pharmacy-led WM programme was the first attempt by Greek pharmacists to expand their role by delivering public health services. The programme was chosen in accordance with the preferences of the Greek pharmacists [23]. It was delivered in Patras, owing to its convenient location and the local knowledge of the researcher and was supported by the obesity statistics in that city.

Direct comparisons with other pharmacy-led weight management service studies (i.e. studies which were conducted in the UK) cannot be made as the demographics, structure of the service (i.e. duration of the programme, number of visits, inclusion criteria) and the situation in each country are not the same. For example, in this study, all participants were White origin. However, in the majority of worldwide studies, the participants were from various ethnic groups such as Black and Asians. In addition, all information provided to participants in this study, including recipes and the food options that they had to choose from, were based on the Mediterranean culture and traditions. Furthermore, all the guidelines they were given, including alcohol consumption, were based on the ESC European guidelines for CVD prevention in clinical practice [10]. An important difference between this WM programme and the UK WM services offered is the use of a structured approach, something that is already implemented by the UK service providers [41]. All pharmacists received the same training, and had a structured way of delivering the programme, including the way in which they collected the results, the visits, as well as the advice given to the participants, which incorporated both verbal and written communication as both were deemed important by interviewed pharmacists in a previous study [23]. Thus, regardless of where participants received the programme, they had the same booklets containing the appropriate information about weight loss and improving their lifestyle.

With regards to the characteristics of the sample population that received the intervention, the majority of the participants were females, a finding that was observed in similar studies [42, 43]. This may be explained by the Poobalan et al. [44] study, which indicated that men do not seek early medical advice compared to women, leading to poorer health outcomes for men. Furthermore, the mean BMI of the female sample population was 30.78 kg/m^2^ and for males was 32.10 kg/m^2^, classifying them as obese class I. This finding is not in line with the national mean BMI for males and females (28 kg/m^2^ and 27 kg/m^2^, respectively), which classify them as overweight.

An interesting finding was that there were more female smokers than males in the sample. According to the WHO, the increase of tobacco smoking in the female adult population can be attributed not just to social factors and females’ increasing economic resources, but also to the tobacco industry’s marketing of cigarettes to women as a symbol of emancipation [45]. Interestingly, the majority of participants who were smokers (75.8% (n = 25/33)) did not want to quit smoking, as they believed that they would gain weight. This agrees with various studies that state that most smokers gain weight when quitting smoking, which can interfere with cessation efforts [46]. Although cessation-related weight gain is modest [46], many smokers report gaining more than 10 kg, placing them at high risk of obesity-related chronic diseases.

At the end, the programme achieved a mean weight loss of 7.73 kg (SD = 2.51) for both genders. Although, direct comparisons cannot be made with other weight management programmes; it is however worth pointing out that in a similar programme (12-week programme) conducted in the UK, the mean weight loss achieved was 2.1 kg in the pharmacy arm (total number of participants = 183) [42]. This programme achieved a mean weight loss in the follow-up period (approximately 9 to 12 months post-programme) of 8.21 kg (SD = 2.72). Conversely, in community pharmacy programmes conducted in the UK, the mean weight loss in pharmacy participants at session 15 (approximately 9 months) was 2 kg [42] and 1.7 kg (1 year) [47], respectively. A study conducted by Harmon et al. [48] in Maricopa County in Phoenix, which enrolled 12 individuals, 11 (92%) of whom completed the programme, achieved a mean weight loss of 5 kg (*P*-value < 0.001), with 45% of individuals losing 5% or more of their weight. Similar to our study, there was also a statistically significant reduction in participants’ BMI and WC [48]. So it was clear that this pharmacy-led weight management programme achieved higher weight loss results compared to other ones. In terms of the secondary outcomes (BMI, BP, WC, AUDIT-C score, Mediterranean diet score), the programme was also successful. It is worth mentioning that the 10-week programme was successful not only in terms of weight loss, but also the BMI classification was improved (*P*-value < 0.001). Interestingly, during the follow-up period, further weight reduction was observed; but participants did not change their BMI category. Each BMI category reflects 14 kg in weight which is beyond 5–10% target weight loss. Therefore, a weight loss outcome is more achievable than a change in BMI category and should be maintained as the primary outcome of future services. Moreover, only 3 participants were classified as hypertensive at the end of the programme compared to 19 originally (*P*-value < 0.001). This is another achievement of this WM programme and it accords with various studies [1, 49, 50], which mentioned that weight loss positively contributes to BP reduction leading to a reduction in CVD risk. Interestingly, the study conducted by Boardman et al. [37] which evaluated a 6 month weight management programme stated that there was no alteration in participants’ BP following a 3 month period but there was after 6 months. This could be attributed to the more than double weight loss achieved (7.73 kg) compared to 3.07 kg observed by Boardman et al [37]. Another factor that may have helped the BP reduction during the programme was the increase in participants’ levels of activity. Chobanian et al. [51] stated that regular physical activity reduces systolic and diastolic BP. It was obvious that during the weight management programme, participants significantly improved their exercise levels (*P*-value < 0.001) and decreased their sedentary lifestyle. The increase in physical activity can help in reducing weight and it can also lead to the prevention of type 2 diabetes and CVD [52]. As stated in the Haskell et al. [53] study increased levels of physical activity should be an integral part of any weight loss option for obese individuals regardless of weight loss goals as it is associated with many CVD benefits [52]. In the study, 83.8% of the participants who reported that they were drinking significantly decreased their AUDIT-C score after the programme’s conclusion (*P*-value < 0.001). Furthermore, through the programme, there was a statistically significant decrease in both male (*P*-value = 0.004) and female (*P*-value < 0.001) participants WHtR, which is an early indicator of CVD risk [9]. There was also an increase in participants’ Mediterranean diet score [(≥ 10) (*n* = 10, 8.5% at initial screening and *n* = 33, 28.2% at the last visit)].

A minority of participants (*n* = 3) did not lose weight. They were offered the programme by three different pharmacists working in different pharmacies who ran the programme successfully with their other participants. For example, one pharmacist had 11 participants, and of those only 1 did not lose weight. Therefore, the lack of change in weight could have been a result of participants’ lack of effort, and their lack of commitment in properly following the advice and support gained from the programme.

The programme was successful, as 114 out of 117 enrolled individuals reached the primary outcome of the programme, which was to achieve a weight reduction of at least 5% of their initial weight. A possible explanation for this success could be the fact that Greek pharmacists chose the WM programme as the first service to deliver by themselves. This means that pharmacists engaged more in delivering the programme, as it was chosen by them as a future need for enhancement of their current role. Another explanation for the programme’s success could be the continuous support that pharmacists had during the programme’s delivery from the lead pharmacist. An additional reason for the programme’s success could be the homogenous population both in terms of ethnic groups and also the fact that individuals with special dietary requirements were excluded; the advice given was applicable to everyone, whereas in other countries such as the UK, advice needs to be tailored, as there are various cultures and different ethnic groups. A further possible reason for the programme’s success is the fact that structured training was offered using role playing, based on participants’ training needs and preferences. The Kocla-Kimble et al. [54] study, which designed an active learning model for pharmacists offering diabetes care, showed an increase in their problem-solving abilities, their communication skills and their self-confidence.

Another interesting observation which make this programme successful was that none of the enrolled participants dropped out of the programme, an achievement that contrasts with other WM programmes. For example, in a study conducted by Nanchahal et al. [55], loss to follow-up was high (43%), similar to that of other weight loss studies in the UK [56, 57]. This could be explained as the length of the programme was concise and the meetings took place biweekly rather than weekly. Another factor that may have played a role was the training that pharmacists received on how to motivate people to make a change. This finding agrees with McCormick et al. [58], who indicated that training can have a “protective” role by minimising participants’ withdrawal from services.

According to the Bush et al. [43] study, a factor that may have affected the relative success of this programme was the measured weight and WC of participants upon their entry onto the programme. It has been identified that people who are classified with high BMIs (≥ 35 kg/m^2^) are more prone to lose more weight and consequently to decrease their BMI categories, compared to those weighing less at the initial screening. In this study 20 participants (17.1%) entered the programme with a BMI ≥ 35 kg/m^2^ with 52.1% of the whole sample being classified as obese at start of programme. This could be a reason why this weight management programme achieved higher weight loss results compared to the pharmacy-led weight management programmes offered elsewhere.

With regards to equipment, measuring tapes were provided to pharmacists. Other than that, each pharmacy had its own set of weighing scales (either electronic or analog) and BP machine (either electronic or manual). This might have acted as a limitation in the accuracy of readings, as not every pharmacy had the same equipment to use in the service. More specifically, measurements obtained might vary between the analog weight scale/manual BP monitor and the electronic ones. Additionally, some participants thought that the area where the meetings took place was not appropriate. A possible solution for this could be the introduction of consultation rooms to pharmacies in Greece, in order to increase privacy and confidentiality when offering public health services, a model that the UK already follows [59]. Although this is a possible solution to overcome this issue, the Weidmann et al. [60] study showed that 47.3% of their sample stated lack of privacy as a barrier in Scottish pharmacies (a country where there are consultation rooms within pharmacies).

In terms of participants’ recruitment, there were variations between pharmacies. This may reflect the confidence in approaching clients, the communication skills and the ability of pharmacists to motivate clients all of which are difficulties already highlighted by pharmacists [23]. Therefore, continuous training should be offered to ensure that pharmacists are always in a position to recruit and offer public health interventions.

In terms of limitations, it must be noted that the participants’ collected data were recorded by pharmacists, and the analysis therefore depended on the accuracy of the recorded data, in particular on the measurements or calculations obtained (in the case of using the paper-based booklets). In addition, the self-reported physical activity level by participants could be another limitation, as the duration might have possibly been overestimated. Participants also self-reported their height, so there may be a difference between BMI measurements due to inaccuracies in the reported heights.

Although the sample size was achieved, it can be argued that it is small for the generalisation of findings across Greece. Therefore, the study needs to be repeated in another city in Greece to ensure its repeatability including broader inclusion criteria such as individuals with special dietary requirements such as vegetarians. Additionally, to implement the WM programme in other areas of Greece, the local pharmaceutical associations should agree their participation and a holistic training should be offered to pharmacists practising in different cities in Greece. Moreover, replication of the programme is very important as the success of the programme should be replicated in both larger and smaller cities rather than Patras to be able to generalise the results. Furthermore, a comparison of the demographics of the participants against the general population who are overweight/obese in Greece could not be performed due to the lack of the availability of this data. Therefore, it is difficult to conclude if the sample is representative of the population under investigation. Lastly, only about 12% of the pharmacists in Patras agreed to take part. This was mainly due to lack of awareness and time challenges. Such limitations need to be considered if this programme is to act as a model for pharmacy public health services.

## Conclusions

Despite the limitations, our findings provide a model for a pharmacy-led public health programme revolving around weight management that can be used as a model for services in the future. The programme consisted of a 10-week intervention which assessed in addition to the weight loss, the BMI, WC, BP, AUDIT-C score and Mediterranean diet score. Thus, it had a more holistic approach. The programme benefited from structured visits and supplementary materials to aid participants and pharmacists alike. All pharmacists were provided with training and the programme was evaluated both qualitatively and quantitatively. The programme had a success rate which supersedes any pharmacy-led programme for weight loss, thus adding to the evidence of pharmacists’ role in public health in general and to the potential role of Greek pharmacists in that regard in particular. Finally, pharmacy-led preventive services advance pharmacists’ clinical role in the community. The success of the pharmacy-led interventions in a national level will add value to pharmacists’ role in addressing obesity as well as it will give them financial incentives for offering such services into their daily practice.

## Data Availability

The authors are happy to share any of the raw materials to any scientist wishing to use them.
